# toxoMine: an integrated omics data warehouse for *Toxoplasma gondii* systems biology research

**DOI:** 10.1093/database/bav066

**Published:** 2015-06-30

**Authors:** David B. Rhee, Matthew McKnight Croken, Kevin R. Shieh, Julie Sullivan, Gos Micklem, Kami Kim, Aaron Golden

**Affiliations:** ^1^Department of Genetics, Albert Einstein College of Medicine, Bronx, NY 10461, USA,; ^2^Department of Medicine,; ^3^Department of Pathology,; ^4^Department of Microbiology & Immunology, Albert Einstein College of Medicine, Bronx, NY 10461, USA,; ^5^Department of Genetics, University of Cambridge, Cambridge CB2 3EH, UK and; ^6^Department of Mathematical Sciences, Yeshiva University, New York, NY 10033, USA

## Abstract

*Toxoplasma gondii* (*T. gondii*) is an obligate intracellular parasite that must monitor for changes in the host environment and respond accordingly; however, it is still not fully known which genetic or epigenetic factors are involved in regulating virulence traits of *T. gondii*. There are on-going efforts to elucidate the mechanisms regulating the stage transition process via the application of high-throughput epigenomics, genomics and proteomics techniques. Given the range of experimental conditions and the typical yield from such high-throughput techniques, a new challenge arises: how to effectively collect, organize and disseminate the generated data for subsequent data analysis. Here, we describe toxoMine, which provides a powerful interface to support sophisticated integrative exploration of high-throughput experimental data and metadata, providing researchers with a more tractable means toward understanding how genetic and/or epigenetic factors play a coordinated role in determining pathogenicity of *T. gondii*. As a data warehouse, toxoMine allows integration of high-throughput data sets with public *T. gondii* data. toxoMine is also able to execute complex queries involving multiple data sets with straightforward user interaction. Furthermore, toxoMine allows users to define their own parameters during the search process that gives users near-limitless search and query capabilities. The interoperability feature also allows users to query and examine data available in other InterMine systems, which would effectively augment the search scope beyond what is available to toxoMine. toxoMine complements the major community database ToxoDB by providing a data warehouse that enables more extensive integrative studies for *T. gondii*. Given all these factors, we believe it will become an indispensable resource to the greater infectious disease research community.

**Database URL:** http://toxomine.org

## Introduction

*Toxoplasma gondii* (*T. gondii*) is an obligate intracellular parasite that causes the disease toxoplasmosis. Its definitive hosts are members of the Felidae family and there is a wide range of intermediate hosts available as it can infect almost all warm-blooded animals. As a waterborne pathogen, it is classified as a Category B Biodefense agent and has been estimated to infect up to a third of the world’s population. As a part of the Apicomplexan parasitic group, *T. gondii* must regularly monitor for changes in the host environment and respond accordingly. It is thought that epigenetic regulation, changes in gene expression and subsequent activation/deactivation of gene networks play a role in the regulation of its pathogenic potential and virulence traits as it adapts to changes in the environment (1, 2). It is still not fully known which genetic and/or epigenetic factors are involved in the aforementioned processes but there are on-going efforts to elucidate the mechanisms regulating the virulence traits of *T. gondii*.

These efforts have been undertaken through a systematic approach of applying high-throughput epigenomics, genomics and proteomics techniques to uncover potential factors regulating stage transition processes (3). For example, Chromatin immunoprecipitation (ChIP) protocols in conjunction with either microarray (ChIP-chip) or high-throughput sequencing (ChIP-seq) technologies is deployed to map protein binding sites and characterize epigenetic signatures of different stages of *T. gondii* (4, 5). Gene expression studies comparing multiple virulence traits to identify key changes in individual gene expression and corresponding gene network patterns are captured using microarray-based or sequencing-based (RNA-seq) technologies (6, 7). Furthermore, protein–protein interaction studies via mass spectrometry are being utilized to identify macromolecular complexes and subsequent protein networks involved in regulating the virulence traits of *T. gondii* (4).

Given the range of experimental conditions and the typical yield from such high-throughput techniques, a new challenge arises: how to effectively collect, organize and disseminate the generated data for subsequent data analysis. Furthermore, not only is there a need for proper data storage and management but also there is an additional need to capture relevant metadata to describe the stored data. Such a need was identified and addressed by members of the modENCODE Project, an initiative devoted to elucidating all genomic functional elements in the model organisms *Caenorhabditis* *elegans* and *Drosophila melanogaster* by means of integrating similarly diverse data sets generated from a battery of molecular assays (8, 9). An infrastructure capable of storing and managing data, as well as cataloging metadata, was developed as part of this project, the modENCODE Data Coordination Center (DCC) (10), which forms an excellent blueprint for the storage, management and curation of such diverse data products based around a fundamentally integrative analytics agenda.

In pursuit of the integrative analytics agenda, the modENCODE consortium adapted a data warehouse strategy, utilizing the InterMine system, an open-source data warehouse framework designed for seamless integration and analysis of complex biological data (11). As a feature-rich framework, one of its focal points is the highly flexible and extensible data model, which allows the incorporation of a wide range of existing biological data with the ability to easily add new data types. This is possible by the use of the Sequence Ontology (SO) framework (12) to connect all data entities within the same structured, and thus ‘searchable’, data space. Whilst SO is based around the consensual definition of genomic sequence features used in biological sequence annotation, the fact that one can express any level of associated biological process or context in relation to the SO schema provides a means to integrate genomic, epigenomic, proteomic, pathway/gene ontology (GO) level, metabolic and experimental metadata entities within the same data model. It also features an exceptionally fast and flexible query engine, which grants the ability to build and add custom queries relatively effortlessly, either using the graphical user interface’s (GUI) querying facility or remotely through the many application programming interfaces (APIs) available. This querying capability is a unique feature that allows users to search and ask questions that encompass many data sets. Furthermore, the InterMine framework comes with innate interoperability to ‘communicate’ with other InterMine-based systems using the InterMOD infrastructure (13). There are a number of InterMine variants already available for many organisms, e.g. FlyMine, YeastMine, TargetMine, MetabolicMine, MitoMiner, INDIGO, RatMine and MouseMine (14–20).

Leveraging the capabilities of the InterMine system, the modENCODE consortium released their own engineered version called modMine (18), which is capable of interacting with vast amounts of data stored in the modENCODE DCC plus data from other third-party databases. Taking a cue from this successful modENCODE initiative, we are emulating a similar approach in our ongoing studies of the developmental and virulence processes associated with *T. gondii*, by means of an integrated systems biology approach to analyse the generated high-throughput experimental data (Chip-chip, ChIP-seq, RNA-seq, proteomics) generated by a collaboration lead by Dr K. Kim here at the Albert Einstein College of Medicine. In this article, we describe toxoMine, our InterMine implementation, which provides the same powerful interface to support sophisticated integrative exploration of high-throughput data and metadata, providing researchers with a more tractable means toward understanding how genetic and/or epigenetic factors play a coordinated role in determining the pathogenicity of *T. gondii*. As a freely available public data warehouse, toxoMine allows us to integrate high-throughput data sets with public *T. gondii* data, such as those currently residing on the community resource ToxoDB (21). Furthermore, interoperability features help users query and examine data available in other InterMine systems, extending the search scope beyond what is available in toxoMine. Our goal is that this approach will provide opportunities for the identification of novel drug targets for *T. gondii* and pave the way for other eukaryotic pathogens. toxoMine complements the major community database ToxoDB by providing a data warehouse that enables more extensive integrative studies for *T. gondii*; therefore, we believe it will become an indispensable resource to the greater infectious disease research community.

In the following sections, we describe the construction of toxoMine. We subsequently describe how access is mediated to this data space and illustrate the capabilities of the system using examples supporting on-going studies of *T. gondii* virulence.

## Building toxomine

As a data warehouse framework, InterMine allows the integration of a wide range of biological data sources by framing its underlying data models on an ontology-based foundation. In particular, a collection of biological models serving as the backbone of the system was assembled by applying terms and relationships using the SO (12). InterMine not only comes with a number of ‘core’ biological data models based on SO terms and relationships that are indispensable, e.g. chromosomes, genes and proteins, but it also has the flexibility to include any other SO terms such as histone binding sites, introns and ncRNA as needed. Building an infrastructure based on a well-known and mature ontological framework allows different data sources to ‘talk’ to one another, leading to smooth integration of disparate data types. More specifically, the integration of equivalent data objects from distinct data sources, e.g. genes and proteins, are facilitated by the unification of objects with a common identifier. For instance, a gene identifier that uniquely describes each gene object is used to integrate genes from multiple data sets. Furthermore, data sources are prioritized to resolve any potential disparities that would have otherwise arisen during the integration process. For example, the definition of a protein short name from two data sources may differ and cause an error during the data integration step, but developers can define which source definition will take precedence.

In addition to providing base data models, InterMine makes it easy to extend existing data models and/or add custom data models when adding data types that do not have definitions in SO. Built on top of this ontology-based and flexible data model paradigm, toxoMine integrates a number of publicly available data sets and high-throughput experiment data sets by extending the ‘core’ data models with the addition of high-throughput experiment data-specific model definitions. For instance, data models uniquely added/extended for toxoMine include but are not limited to project, experiment, submission, microarray, toxoplasma mutant and antibody (Supplementary Table S1). Briefly, project, experiment and submission data types are extended from the ‘data set’ data model, which is one of the ‘core’ data types that include some of the data attributes needed to describe the aforementioned data types. Furthermore, additional data attributes can be declared if warranted. For instance, the embargo date attribute is unique to the submission data type thus it is added separately. High-throughput omics-specific data types such as microarray, toxoplasma mutant and antibody are extended from a newly created data type called submission property, which itself is extended from the ‘InterMineObject’ data type—a ‘core’ data type that serves as the base of all InterMine objects. For outputs of high-throughput data sets such as ChIP-chip, SO term ‘binding site’ is used and therefore only needs to be extended by declaring and linking to a corresponding submission data object via foreign key. This means that, once all necessary data types are declared, we simply add additional attributes that can be used to link data types to other existing data types or newly created data types by adding one or many foreign keys that point to one another.

### Integration of publicly available *T. gondii* data sets

The integration process begins by loading genome sequences and annotation data sets. The genomic data for *T. gondii* were obtained from ToxoDB (21). This process is automated by the use of a configurable data downloader and data parser that comes with InterMine. Release 11.0 for three of the most widely used strains, ME49, GT1 and VEG was incorporated into the database in this way. To complement the genome annotation data, we also integrated transcription start site (TSS) region data from Yamagishi *et al.* (22). Subsequently, protein and protein domain data were integrated into toxoMine using a similar approach from Uniprot and InterPro, respectively (23, 24). To support comparative analysis, homolog data were integrated from the OrthoMCL database (25) and GO data from the GO consortium database (26). Additionally, toxoMine was loaded with publication data from the PubMed database to support publication-related searches and analysis. The summary of all publicly available data sets loaded into toxoMine can be found in the ‘Data Sources’ tab.

### Integration of high-throughput experiment data

**Figure 1.**
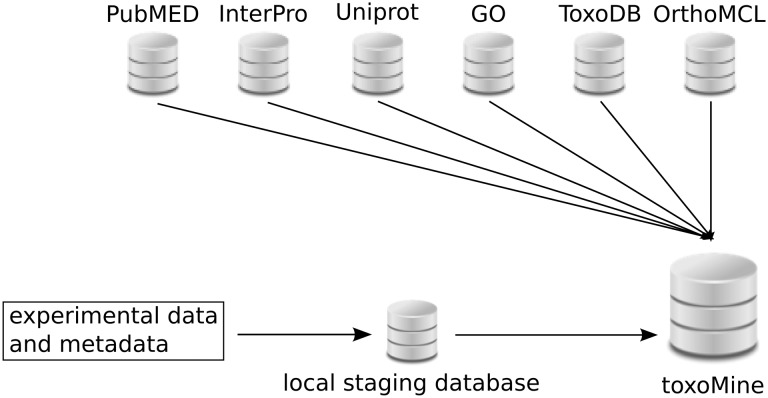
Layout of toxoMine setup and building process. All high-throughput experimental data and metadata is populated in the local PostgreSQL database for staging purposes. Subsequently, organized data is integrated into toxoMine along with data from third-party databases.

All high-throughput experiment data and metadata are loaded into the local PostgreSQL database before integration into toxoMine (Figure 1). This local database serves as a staging area to store and organize data and metadata generated from a number of high-throughput biological assays. The general idea and organization of this local database is very similar to the well-established modENCODE DCC, in that both databases host and curate metadata which describe experiments and related submissions in addition to hosting resulting data from a variety of conducted biological assays. More detailed information about the modENCODE DCC has already been published (10). All data in the local staging database are organized into projects that describe an overall research goal (Supplementary Figure S1). These projects can be maintained by a single principal investigator (PI) or multiple PIs. A single project can have multiple experiments, which are an instance of different types of typical high-throughput biological assays or experiment techniques. Subsequently, each experiment coordinates related submissions together. An individual submission is a container of metadata, experimental protocols applied and resulting data from a particular assay testing a number of experimental conditions and factors such as growth conditions, temperature, antibody and/or strains. To accommodate the export and import process from the local database to toxoMine, a custom parser was written to query and retrieve applicable metadata from the local database and load it into toxoMine via InterMine Extensible Markup Language (XML) format. For the resulting biological features data, a custom parser was written to handle the importation process using a general feature format (GFF3) file (http://www.sequenceontology.org/gff3.shtml). In addition, a gene-list data model was created, along with an appropriate data parser, to incorporate lists of genes into the database.

We illustrate the project-oriented organization in both local staging database and toxoMine by describing a specific project, ‘GCN5b’, which is a project available under the ‘Browse all high-throughput data’’ section on the front page. The ‘GCN5b’ project is a recently published work characterizing GCN5b’s role as an essential histone acetylase regulating gene expression during the *Toxoplasma* lytic cycle (4). This project has two experiments loaded into toxoMine—‘Identifying genome-wide binding sites for GCN5b’ and ‘Identifying GCN5b protein–protein interaction partners’. The first experiment contains submissions that are biological replicates of three ChIP-chip experiments to identify genome-wide localization of GCN5b—over 36 900 identified protein binding sites were loaded into toxoMine as biological features. The latter experiment consists of two replicates of protein–protein interaction experiments via mass spectrometry, and over 50 identified interacting partners were loaded into toxoMine as a gene list.

### Post-processing and deployment

When the data integration step is finished, both mandatory and optional post-processing steps are required. These post-processing steps are discussed in more detail elsewhere (11). Briefly, establishing relationships between object entities, which were not otherwise possible during the data integration step, is usually performed in the post-processing phase. Additionally, creating an attribute index and Apache Lucene (open-source index search engine) based keyword index for webapp usage is also executed during the post-processing phase. Depending on the size of data sets, integrating and post-processing steps require significant amounts of computation time and resources, which can be taxing on the hardware. It is recommended that two independent build/production systems are used alternately for building and deploying an InterMine-based system. At the moment, toxoMine uses two Linux servers interchangeably, each with 12-core Intel processors, 96 GB RAM and 4 TB of RAID 10 hard drive space, as build and production systems. Upon building the database, a toxoMine webapp is deployed with Apache tomcat (http://tomcat.apache.org).

## Accessing toxomine

The toxoMine system comes with a user-friendly web interface and powerful tools that give users the capability and flexibility to explore and interrogate data and metadata available using a number of different approaches. By providing simple data browsing features in addition to sophisticated query engine technology, among other functionalities, toxoMine enables users to perform anything from rudimentary data exploration to highly complex data examination. For the latter, users can simply utilize already available ‘templates’ (pre-defined queries within the web interface) or construct custom queries using the web interface’s query builder. This functionality gives users the flexibility and power to ask relevant questions without having the requisite informatics proficiency often needed to do so, implicitly supporting reproducible research as a result. For those with programming skills, APIs in multiple common programming languages can be used to run custom searches across the database. More detailed descriptions of InterMine’s data querying and analysis capabilities have been discussed elsewhere (16). Here, we will focus on toxoMine’s features and its practical usage through examples that demonstrate how toxoMine is a unique and powerful tool for the greater research community.

### Browsing

The list of data sources used to populate the toxoMine database is accessible via the ‘Data Sources’ tab at the top of the homepage. Once there, a user can browse any data category by simply clicking the appropriate icon on the left side. For instance, one can navigate all high-throughput experimental data and metadata by selecting the appropriate icon. This will list all of the projects, experiments, submissions, related data and metadata uploaded from the local staging database. Each individual data object, e.g. project and submission, have a report page fully customized to display pertinent information. For example, clicking on the ‘Identifying genome-wide binding sites for GCN5b’ experiment under the ‘GCN5b’ project will load detailed information about this particular experiment. Once there, the experiment report page shows that, among other things, this experiment consists of several submissions and also provides links to their individual report pages.

**Figure 2.**
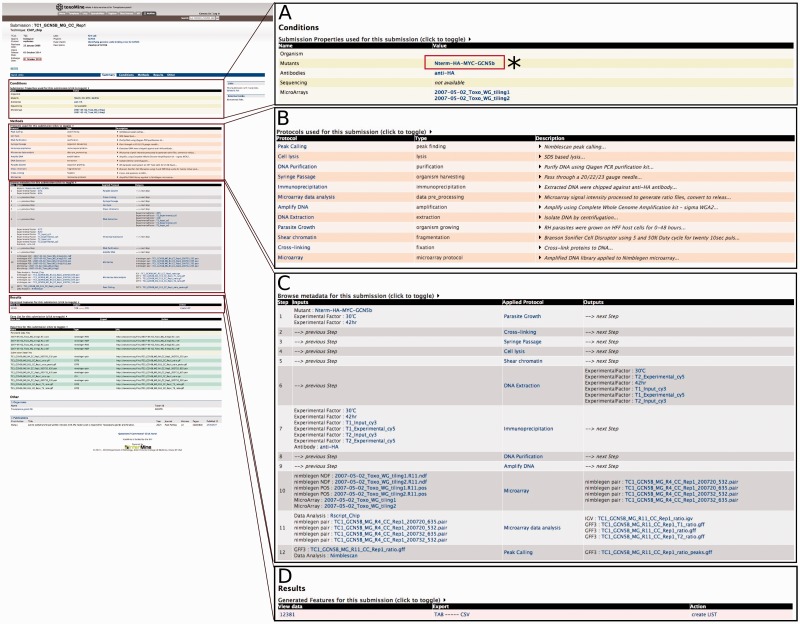
An example of the Report page. Here, we focus on submission ‘TC1’ to illustrate that a customized report page contains information from a range of associated data sets. (A) (*) Users can click on the Mutants link to seek additional experiments or projects that used this mutant. This will lead users to a report page for that particular data entry. (B) List of all protocols involved in this submission with type and description of protocol displayed. (C) Report page lists all of the data generated and detailed applied protocol information including antibody, mutant and the type of software used to generate the data. (D) The resulting data for the submission can be exported into a number of different formats, e.g. TAB and CSV. Additionally, result data can be exported as a ‘list’ for further analysis.

The interconnected nature of data object entities to any related data and metadata allows users to simply select any report page of interest as a starting point for further exploration. For example, a user can select one of the submission links from the previous experiment, e.g. submission ‘TC1’, to navigate to that submission’s report page for greater detail. The submission report page not only lists all of the data generated, but it also presents applied protocols with enough detail to replicate the given experiment. These protocol details include, but are not limited to, a brief description of the protocol, antibody and mutant used, and the type of software (including parameters) used to generate and analyse the data. To explore further, users can continue to other report pages of interest. For example, a user can click on the ‘Nterm-HA-MYC-GCN5b’ Mutants link under the Conditions section from the submission ‘TC1’ report page to seek additional experiments or projects that used this mutant (Figure 2).

### Data querying

**Figure 3.**
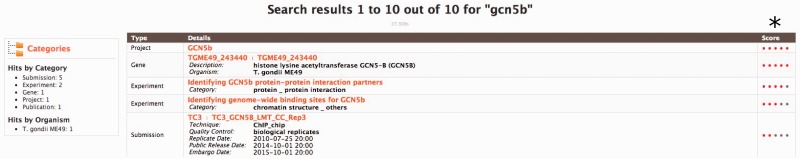
An example of using the ‘Keyword Search’ tool to query toxoMine. We searched toxoMine using the keyword ‘gcn5b’ to find all relevant data objects. The search result shows that a total of 10 data objects contained the search term. (*) The results are sorted based on a score indicative of how closely the keyword correlates to the saved search index.

To help users quickly find the most relevant data, toxoMine comes with the ‘Keyword Search’ tool that allows users to search for data using terms of interest. These terms include but are not limited to Gene symbols, PubMed identifiers, Protein Accession identifiers or any keywords such as ‘tagged’ for antibodies. For example, searching the keyword ‘gcn5b’ brings up all data objects with the keyword ‘gcn5b’ in it. The relevance of the keyword related to the returned objects is highlighted as a score on the right side of the returned results (Figure 3).

**Figure 4.**
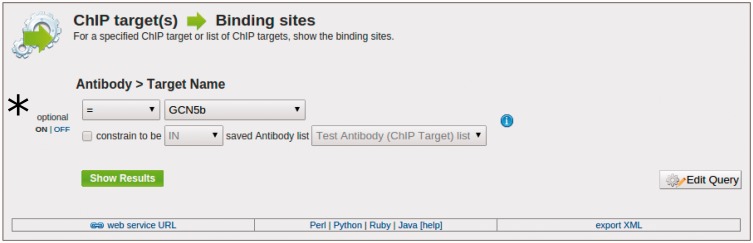
An example of the ‘Templates’ search tool. Here, we demonstrate the usage of the ‘ChIP target(s) → Binding sites’ template to find all of the binding sites that are targeted by a specific antibody’s ChIP target. (*) Users have the option to choose the antibody’s target name, provide a list of ChIP targets, or turn the filter off altogether to broaden the search criteria.

The toxoMine system also provides a library of ‘templates’ that users can employ to perform more sophisticated searches across the database. As previously mentioned, ‘templates’ are a user-friendly web-interface to pre-defined database queries that consist of clean HTML forms with configurable filters for a more refined and targeted searching experience. There are a number of ‘templates’ already available in toxoMine and users can construct, save and share their own. For example, the ‘ChIP target(s) → Binding sites’ template can be used to find all of the sites that are bound by a specific antibody’s ChIP target. Here, users have the option to choose the antibody’s target name, provide a list of ChIP targets or turn the filter off altogether for broader searching criteria (Figure 4).

The ‘templates’ are not limited to simple queries that only involve a few data objects. The ‘templates’ are also capable of performing more complex queries that involve many interconnected data objects. For instance, with the ‘ChIP target(s) + Gene(s) → Binding sites in Gene flanking region’ template, a user can find binding sites that are targeted by a specific antibody’s ChIP target with the condition that binding sites are flanked by a gene or genes of interest. In this example, a user can also specify the direction and distance of the gene flanking region, and the user is not limited to only one gene but has the option to use a saved ‘list’ of genes from the ‘Lists’ feature (Lists features and analysis will be discussed in a later section). Regardless of the type of ‘templates’ or data objects involved, the results are always returned in a table format that the user can interact with. Users can perform cursory analysis with spreadsheet-like functionality, e.g. column sorting, and have the option to choose which columns to display before exporting the table into a number of different file formats for further analysis. The output formats include, but are not limited to, tab-separated (TAB), comma-separated (CSV), XML and GFF format. In addition, a user can export the table into a ‘list’ and perform ‘list analysis’.

**Figure 5.**
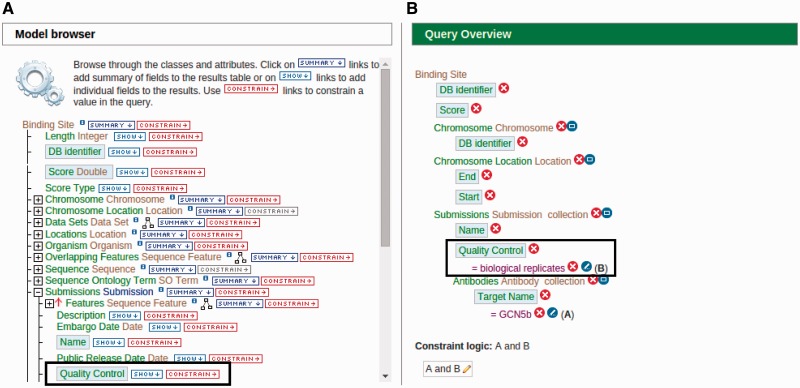
An example of modifying a template using the ‘QueryBuilder’ tool. An existing ‘ChIP target(s) → Binding sites’ template is being modified to display an additional attribute and limit the output by applying a filter. (A) Using the ‘Model browser’ on the left, we enable the attribute ‘quality control’ by clicking the ‘SHOW’ button. This will display quality control attributes in the final result. We can also limit the results by applying a constraint value of ‘biological replicates’ to the quality control attribute by clicking the ‘CONSTRAIN’ button. (B) The ‘Query Overview’ to the right shows that the filter ‘biological replicates’ has been applied to the attribute quality control.

In addition to using a library of existing ‘templates’, users can modify existing ‘templates’ or create a new query template using a tool called ‘QueryBuilder’ for a more personalized querying experience. One of the benefits of using this tool is that it does not require any high-level informatics skills to execute a database query on a complex data set that would have otherwise been difficult for a bench scientist to perform. This is, in part, due to QueryBuilder’s intuitive user interface that is visually simple and easy to use. The ‘QueryBuilder’ tool allows users to browse through the integrated data model and visually construct a query by selecting data attributes of interest to display while defining parameters to serve as filters. For example, users can modify the existing ‘ChIP target(s) → Binding sites’ template by adding the quality control attribute to be displayed and limit the output by only selecting results that have ‘biological replicates’ as its quality control attribute (Figure 5). Users have the option to make modified or custom-built templates available to the public if the template is deemed useful and has potential to be used by others.

### Regions

**Figure 6.**
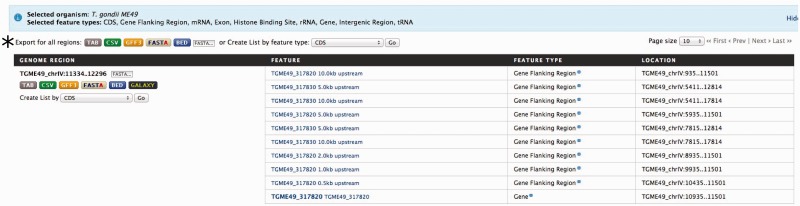
An example of the ‘Regions’ tool showing the result of all applicable genomic features. Here, we provided the chromosome coordinates of 10 MB, both directions, flanking the gene ‘TGME49_317280’. The results are presented as a table with each selected genomic feature listed with links to individual report pages for more details. (*) The results can be transformed into a ‘list’ for further analysis or downloaded into a number of different formats, which include TAB, CSV, GFF3, FASTA and BED.

With the number of high-throughput experiments characterizing genomic features across the entire genome increasing, e.g. histone binding sites and transcription factor binding sites, one of the most common exploratory analyses is examining for overlapping features, or lack-thereof, in specific regions of interest. This is usually done by first downloading all available genomic features, followed by manually parsing out the regions of interest using some sort of custom written script or third-party software. Alternatively, toxoMine provides a tool called ‘Regions’ that streamlines the genomic features search process. The ‘Regions’ tool comes with a user-friendly interface that lets users upload a set of chromosome coordinates and quickly retrieve a list of genomics features available in toxoMine. Furthermore, users can choose which genomic features, e.g. genes, exons and histone binding sites, to include in the results. For example, a user can search for all of the genomic features near the gene ‘TGME49_317280’ by providing the chromosome coordinates flanking the gene. Additionally, users can extend the search parameter by adding flanking regions of up to 10 megabases (MB) in both directions. The results are presented as a table with selected genomic features, which can be downloaded or transformed into a ‘list’ for further analysis (Figure 6).

### Lists

**Figure 7.**
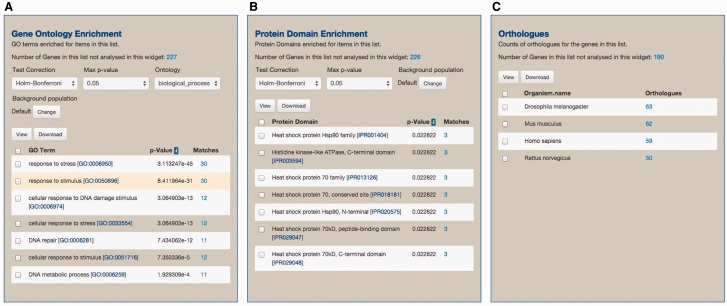
An example of the ‘List analysis’ page showing outputs of graphical and statistical analysis widgets. We used 30 genes annotated with the GO term ‘response to stress’ along with 270 randomly selected genes to generate the result. (A) As anticipated, GO enrichment analysis shows that the GO term ‘response to stress’, among other GO terms, is statistically significant. (B) Protein Domain enrichment analysis shows that many enriched domains were found including ‘Heat shock protein HSP90 family’ domain. (C) Numerous orthologs, including 59 Human genes, were found among 300 uploaded genes. These orthologous gene lists can be used for further analysis via MOD compliant InterMine variants.

As part of the standard web features included in the InterMine framework, toxoMine provides a tool called ‘Lists’ that allows users to create and/or manipulate existing lists of data objects in the database. There are a number of existing lists in toxoMine, e.g. ‘GCN5b protein interacting partners’, which users can readily consume for analysis. Additionally, toxoMine allows users to create their own tailored list using a variety of approaches. These approaches include modifying existing lists by combining with, or subtracting from, another list in toxoMine, creating a list from search results, creating a list from result data found in submissions or uploading a list from external sources (Supplementary Figure S2). When a new list is created from an external source, the identity of uploaded data gets resolved automatically if applicable. This means that users can upload identifiers from an older version, which are converted into the most current release of identifiers. For example, old gene identifier ‘TGME49_043440’ gets automatically converted into the updated gene identifier ‘TGME49_243440’ during the upload process. When the loading process is finished, the ‘Lists’ function generates a ‘List analysis’ page that provides a data table along with summary statistics befitting the given list. For example, we uploaded 30 genes annotated with the GO term ‘response to stress’ [GO:0006950] along with 270 randomly selected genes to toxoMine. Upon completion, the ‘List analysis’ page generated a summary of statistics, e.g. GO enrichment analysis, which characterizes the loaded list. The results show that, as expected in GO enrichment analysis, the ‘response to stress’ among other GO terms, is statistically significant (Figure 7).

## Demonstration

To demonstrate the potential usage of toxoMine, we used one of our unpublished ChIP-chip binding site data for TATA-box binding protein TBP1 (collaborative project between Kim and Sullivan Lab). For this demonstration, we only used chromosomes TGME49_chrIa, TGME49_chrIb and TGME49_chrII data (total of 2188 binding sites) and the data were formatted into a positional format required by the ‘Region’ tool (Supplementary Table S2). To start, we loaded the list into the ‘Region’ tool with the ‘TSS Region’ feature toggled on and all other features turned off. Additionally, we selected the option to find sequence features (TSS) that are within 1 kb of the binding sites. Once the list was uploaded and processed, it returned 401 unique TSS regions that are proximal to the chromosomal positions from the uploaded list. Here, we had the option to save the result as a text file and continue our analysis elsewhere or create a list, which we did. Using a template called ‘TSS region(s) + Flanking region → Genes’, we queried for all genes downstream of the newly created list of 401 TSS regions. In this case, we searched for genes 1 kb downstream of the given list of 401 TSS regions and saved the result as a new gene list with 249 unique genes. We further filtered our new gene list against a known gene set previously uploaded to toxoMine called ‘Phosphoproteome’. After filtering the list, 116 genes remained. Once our gene list was filtered against the known gene set, we performed further analysis with the remaining gene list. In this instance, we used a template called ‘Gene(s) → GO Terms’ to query and found 228 related GO terms. Likewise, we converted the gene list into a list of proteins by clicking on the Protein link at the top right corner of toxoMine. With this new list, we were able to apply protein related templates such as ‘Protein(s) → Protein Domains’. Applying the protein template returned 207 domains. Furthermore, we applied the template ‘Gene(s) + Flanking region → Specific overlapping features’ with ‘histone_binding_site’ as the SO term name to query the 1 kb upstream region of our 116 genes to find all histone binding sites. This query resulted in 6533 unique binding sites that included GCN5b and H3K4me3 histone modifications. One of the great advantages of the InterMine platform is its intrinsic interoperability between different instances, permitting the user to export and explore lists of homologs derived from one instance to another. To demonstrate this capability, we exported the list of homologs found in the list of 116 genes to one of many InterMine instances available, modMine, by utilizing the InterMOD functionality. This query resulted in a number of enrichment analyses from a selected model organism that could potentially be useful and pertinent to research at hand, particularly if the comparison model organism was better annotated. Lastly, we saved our list of 116 genes and made it public by adding a [im:public] tag after logging into toxoMine. This allowed us to share this list for querying purposes with everyone and access the list again anytime in the future (Supplementary Figure S3).

Although the ‘QueryBuilder’ tool can be used to build and execute more customized queries in toxoMine, we relied on a combination of available ‘Templates’, ‘Lists’ and ‘Region’ tools to demonstrate the potential usage of toxoMine. We hope this example illustrated the flexibility and power that toxoMine affords users: the ability to ask relevant questions and quickly find what they are looking for without the bioinformatics expertise often required.

## Additional features

### InterMOD

The InterMOD consortium is working to develop a common model organism database (MOD) infrastructure to facilitate knowledge discovery from insights gathered from a number of key model organisms and to promote cross-species research (13). The InterMOD infrastructure gives all InterMine-based systems the ability to interact with each other via homologs. This means by integrating a homology data set from the OrthoMCL database, toxoMine has the capacity to send cross-species queries to other InterMOD compliant systems to explore gene functions beyond what is available to toxoMine. For instance, using a list of candidate genes with homologs, users can click on one of the MOD links on the ‘links to other mines’ page to send queries to that specific MOD for further analysis.

### MyMine

The toxoMine system comes with a useful feature that allows users to save all queries, templates and lists for future toxoMine sessions. This function can be found in the ‘MyMine’ tab at the top of the home page and allows users to create a private workspace to personalize their toxoMine experience. Users also have the option to share any saved queries, templates and lists with other toxoMine users through ‘MyMine’. This feature has an additional benefit of allowing groups of users in a consortium to keep a record of session interactions, and consequently, fulfill the ideals of a fully reproducible research paradigm.

### Web services

toxoMine provides direct access to the database via HTTP web services, which means that anyone can directly interface and programmatically query the toxoMine database. Users can download web service client libraries from standard repositories appropriate for the language of their choice (27). These repositories include CPAN (http://www.cpan.org) for PERL, PyPi (http://pypi.python.org) for Python and Rubygems (http://rubygems.org) for Ruby. Furthermore, the web service client libraries for Java and JavaScript are freely available and hosted by InterMine on their website.

In addition to providing a direct access to the database, toxoMine allows web-enabled services or sites to link their data directly to toxoMine’s report pages and ‘Lists’ pages. This functionality allows other sites such as ToxoDB to not only link individual genes but also lists of genes back to toxoMine. For instance, the following link will send a query to toxoMine, which will display the ‘Lists’ page with given list of genes (http://toxomine.org/beta/portal.do?externalids=TGME49_210300,TGME49_221240,TGME49_222940&class=Gene). More information on this topic can be found in InterMine’s documentation on their website (http://intermine.readthedocs.org). Also, toxoMine links all appropriate data, e.g. genes, back to its original data source for more information.

## Uploading to toxomine

All high-throughput experimental data and metadata are loaded into the local staging database before integration into toxoMine. For this part of the process, we employ a modified version of the BIR-TAB (Biological Investigation Reporting Tab-delimited) formatted files used at modENCODE DCC (10) to ensure consistency and accuracy. This is applied to all data and metadata submitted to the local staging database. More detailed information about the BIR-TAB format is published elsewhere (10) and is also available at the modENCODE DCC website (http://modENCODE.org). Briefly, BIR-TAB file is a tab-delimited formatted file designed to acquire details about the overall project in addition to capturing the entire processes of the experiment. The consistency and accuracy of data and metadata is guaranteed by enforcing users to follow strict guidelines for completing the BIR-TAB formatted files for submission. For instance, users must abide by rules set for each of the fields, as there are required fields and controlled vocabulary that must be strictly followed.

When submitting to the local staging database, users must submit four BIR-TAB formatted files in addition to output files of omics data sets. The required four BIR-TAB files for submission to the local staging database are as follows: a Project Experiment Submission File (PESF), an Experimental Parameters File (EPF), a Protocols File (PF) and an Applied PF (APF). The PESF contains pertinent information and describes multiple submission data types/objects that are linked to a single experiment and ultimately point to a single project. The EPF contains information about the experimental parameters such as microarray and antibody used for that particular submitted experiment. The PF describes protocols used and an APF details the procedure as well as steps taken with all of the inputs and outputs of each protocol applied for the submitted experiment. As for the resulting output files for omics data sets, users must also follow a guideline when submitting to the local staging database. For instance, outputs of ChIP-chip and ChIP-seq data sets must be in GFF3 format and the output of gene lists must be in a tab-delimited text file format. Once submitted, a custom parser validates the submitted data and metadata and loads it to the local staging database.

We encourage anyone with *T. gondii* omics data to participate and provide their data for upload to toxoMine. Users who are interested in participating should visit our website and consult available documentation and examples for more information as we are continually updating the upload procedure and information on our website to accommodate new data sets and making improvements based on user feedback.

## Conclusions and future plans

As a data warehouse, we developed toxoMine to operate as a linchpin that brings together high-throughput experimental data with many publicly available *T. gondii* data sets. toxoMine has been created with the user in mind; by integrating all of the available data sources, toxoMine is able to execute complex queries involving multiple data sets with straightforward user interaction. Furthermore, toxoMine allows users to define their own parameters during the search process that gives users near-limitless search and query capabilities. We believe toxoMine can serve as an excellent integrative system, but certainly not as the authoritative ‘portal’ encompassing all *T. gondii* related omics data—its interoperable design and inherent flexibility complements the other more extensive data resources, most particularly the definitive ToxoDB (21).

We are continuously adding more data as they become available in addition to adding more features to toxoMine as requested from users. Additionally, expression data for RNA-seq data along with appropriate analysis widgets will be incorporated in the near future. A plan is in place to incorporate proteomics data based on the data types developed for MitoMiner (17), another InterMine variant dedicated to mitochondrial proteomics data for a wide range of organisms. To that end, we are working on the submission requirements pertaining to raw data format and a matching parser. We also plan to add related host data, e.g. human and mouse, as host–parasite research is an active area of interest and will continue to be for the foreseeable future. With MOD interoperability, we feel toxoMine can help bridge host–parasite research and will be an asset to the greater research community. Furthermore, we plan to add an ability for toxoMine to ‘talk’ to InterMine variants not including MOD infrastructure, e.g. TargetMine, to aid in drug discovery related research.

## Supplementary Data

Supplementary data are available at *Database* Online.

Supplementary Data
