# Supplementary material for: toxoMine: an integrated omics data warehouse for Toxoplasma gondii systems biology research
Source: Database (Oxford). 2015 Jun 30;2015:bav066. doi: 10.1093/database/bav066 (PMC4485433; doi:10.1093/database/bav066)
Supplement: Supplementary Data [file supp_2015_bav066_index.html]

toxoMine: an integrated omics data warehouse for Toxoplasma gondii systems biology research — Supplementary Data 

# toxoMine: an integrated omics data warehouse for *Toxoplasma gondii* systems biology research

## Supplementary Data

files

- Supplementary Data - zip file
