# Supplementary material for: toxoMine: an integrated omics data warehouse for Toxoplasma gondii systems biology research
Source: Database (Oxford). 2015 Jun 30;2015:bav066. doi: 10.1093/database/bav066 (PMC4485433; doi:10.1093/database/bav066)
Supplement: Supplementary Data [file supp_bav066_suppl_data.zip › New Microsoft Office Word Document.docx]

**Supplemental Figure 1. Simplified schema of local staging database.** All data in the local staging database is organized into projects. A single project can have multiple experiments, which are each an instance of different types of typical high-throughput biological assays. Each experiment coordinates related submissions together, where an individual submission serves as a container of metadata, experimental protocols applied, and resulting data from a particular assay testing a number of experimental conditions.

**Supplemental Figure 2. Generating a list.** List can be prepared using a variety of approaches. (A) Modifying existing lists by combining with, or subtracting from, another list in toxoMine. (B) Creating a list from search results. (C) Creating a list from result data found in submissions. (D) Uploading a list from external sources.

**Supplemental Figure 3. Demonstration of potential usage of toxoMine in steps.** (1) We loaded the TATA-box binding protein TBP1 binding site data into the 'Region' tool with options to find Transcription Start Site (TSS) regions that are 1kb within the binding sites. (2 & 3) The ‘Region’ tool returned 401 unique TSS regions that are proximal to uploaded binding sites. Subsequently, we saved the returned table as a list. (4) Using a template called ‘TSS region(s) + Flanking region 🡺 Gene’, we queried for all genes 1kb downstream of a newly created list of 401 TSS regions. (5) We saved the result as a new gene list with 249 unique genes. (6) We filtered our new gene list against a gene list available in toxoMine called ‘Phosphoproteome’. (7) The result shows there are 116 genes remaining after applying the filter. (8) We used a template called ‘Gene(s) 🡺 GO Terms’ to query for GO terms for the list of 116 genes. (9) Our results show that there are 228 related GO terms for the list of 116 genes. (10) We converted the gene list into a list of proteins by clicking on the Protein link at the top right corner of toxoMine. (11) We saved the resulting 116 proteins as a list. (12) Newly created list of proteins. (13) We applied the template ‘Protein(s) 🡺 Protein Domains’ to query for all protein domains for the list of 116 proteins. (14) The result shows that applying the protein template returned 207 domains. (15) We applied the template ‘Gene(s) + Flanking region 🡺 Specific overlapping features’ to query the 1kb upstream region of the 116 genes list to find all histone binding sites. (16) The result shows that there are 6,533 unique binding sites including GCN5b and H3K4me3 histone. (17) The ‘Lists Analysis’ page shows that the list of 116 genes has homologs from other model organisms. We clicked on one of the links, modMine, to export the list of homologs for further analysis. (18) The modMine ‘List Analysis’ page shows information that could potentially be useful and pertinent for research hand that are otherwise unavailable in toxoMine. (19) We made the list of 116 genes public by adding a [im:public] tag. (20) The ‘Lists’ page shows that the list of 116 genes is visible to everyone.
