# Supplementary figures and images for: toxoMine: an integrated omics data warehouse for Toxoplasma gondii systems biology research
Source: Database (Oxford). 2015 Jun 30;2015:bav066. doi: 10.1093/database/bav066 (PMC4485433; doi:10.1093/database/bav066)

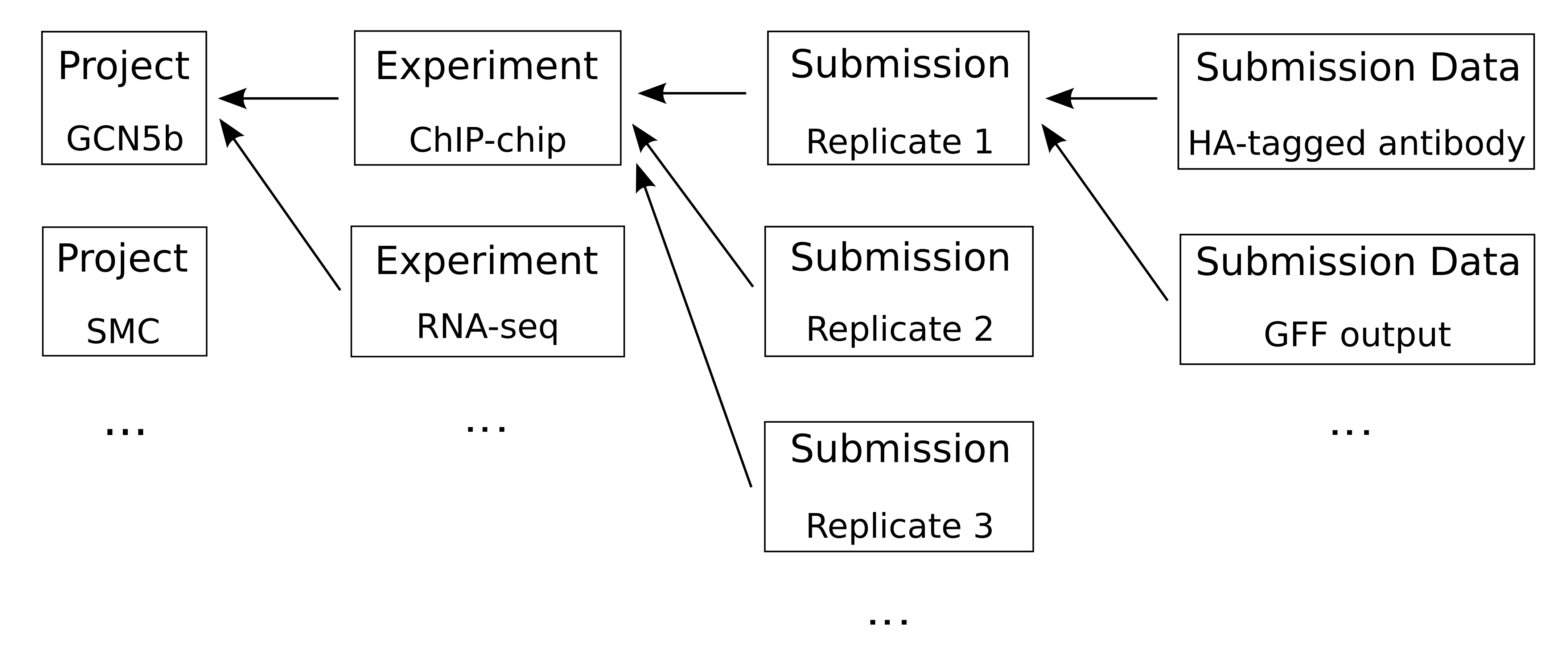

Supplement: Supplementary Data [file supp_bav066_suppl_data.zip › Supplemental_Fig_1.png]

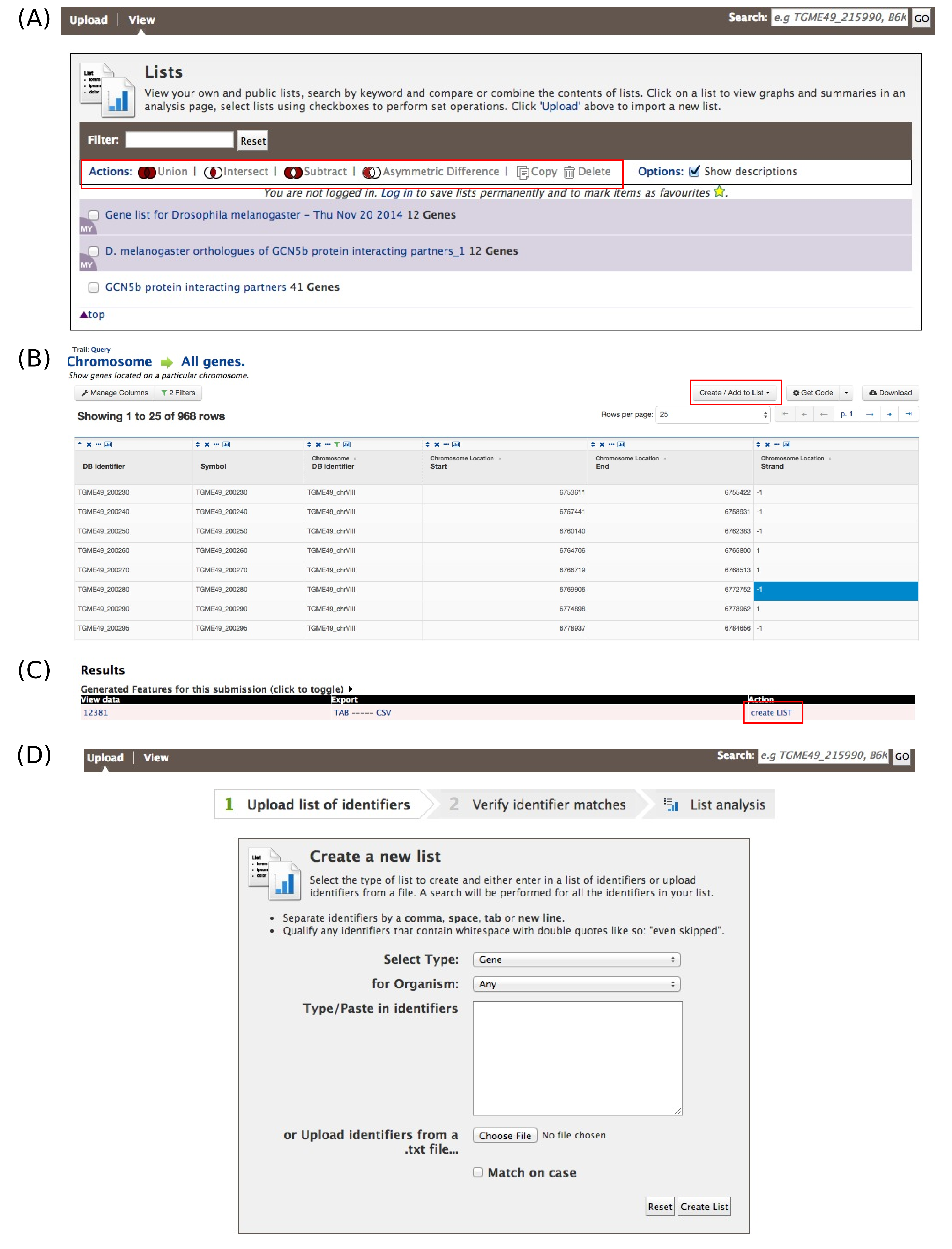

Supplement: Supplementary Data [file supp_bav066_suppl_data.zip › Supplemental_Fig_2.png]
