# Supplementary material for: toxoMine: an integrated omics data warehouse for Toxoplasma gondii systems biology research
Source: Database (Oxford). 2015 Jun 30;2015:bav066. doi: 10.1093/database/bav066 (PMC4485433; doi:10.1093/database/bav066)
Supplement: Supplementary Data [file supp_bav066_suppl_data.zip › Supplemental_Table_1.doc]

**Supplemental Table 1. Data Types in toxoMine**

| **Data Type** | **Description** |
| --- | --- |
| **Applied Protocol** | (Omics) Container of Protocols and Inputs and Outputs |
| **Author** | (Literature) Name of author |
| **BioEntity** | (InterMine) Base class of all biological features |
| **cDNA** | (Genomics) Sequence Ontology term |
| **Protein** | (Proteins) Protein information |
| **Protein Domain** | (Proteins) Protein domain information |
| **Sequence Collection** | (Genomics) Sequence Ontology term |
| **Sequence Feature** | (Genomics) Sequence Ontology term |
| **Binding Site** | (Genomics) Sequence Ontology term |
| **Protein Binding Site** | (Genomics) Sequence Ontology term |
| **Histone Binding Site** | (Genomics) Sequence Ontology term |
| **Insulator Binding Site** | (Genomics) Sequence Ontology term |
| **TF Binding Site** | (Genomics) Sequence Ontology term |
| **CDS** | (Genomics) Sequence Ontology term |
| **Chromosome** | (Genomics) Sequence Ontology term |
| **Exon** | (Genomics) Sequence Ontology term |
| **Gene** | (Genomics) Sequence Ontology term |
| **Gene Flanking Region** | (InterMine) Object that contains all sequence features flanking a gene |
| **Intergenic Region** | (Genomics) Sequence Ontology term |
| **Intron** | (Genomics) Sequence Ontology term |
| **Regulatory Region** | (Genomics) Sequence Ontology term |
| **Enhancer** | (Genomics) Sequence Ontology term |
| **Reverse Primer** | (Genomics) Sequence Ontology term |
| **Transcript** | (Genomics) Sequence Ontology term |
| **Mature Transcript** | (Genomics) Sequence Ontology term |
| **mRNA** | (Genomics) Sequence Ontology term |
| **ncRNA** | (Genomics) Sequence Ontology term |
| **miRNA** | (Genomics) Sequence Ontology term |
| **rRNA** | (Genomics) Sequence Ontology term |
| **tRNA** | (Genomics) Sequence Ontology term |
| **TSS** | (Genomics) Sequence Ontology term |
| **TSS Region** | (Genomics) Sequence Ontology term |
| **UTR** | (Genomics) Sequence Ontology term |
| **Five Prime UTR** | (Genomics) Sequence Ontology term |
| **Three Prime UTR** | (Genomics) Sequence Ontology term |
| **Comments** | (General) Comments |
| **Components** | (Proteins) Protein component |
| **Cross References** | (Ontology) xrefs |
| **Data Set** | (InterMine) Container of data |
| **Project** | (Omics) Project information and container of experiments |
| **Experiment** | (Omics) Experiment information and container of submissions |
| **Submission** | (Omics) Submission information and container of protocols and results |
| **Data Source** | (InterMine) Source of data |
| **Lab** | (Omics) Lab information |
| **EC Number** | (Proteins) Protein EC number |
| **GO Evidence** | (GO) GO evidence |
| **GO Evidence Code** | (GO) GO evidence code |
| **Gene List** | (Omics) List of genes |
| **Homologues** | (Comparative Genomics) Homologues information – orthologues and paralogues |
| **Location** | (InterMine) Location infortaion – e.g. chromosome start and end location |
| **Mesh Terms** | (Literatures) Literature MeSH terms |
| **Ontology** | (Ontology) Ontology source |
| **Ontology Annotation** | (Ontology) Ontology annotations |
| **GO Annotation** | (GO) GO annotations |
| **Ontology Relation** | (Ontology) Ontology relationship |
| **Ontology Term** | (Ontology) Ontology terms |
| **GO Term** | (GO) GO terms |
| **SO Term** | (Ontology) Sequence Ontology terms |
| **Ontology Term Synonym** | (Ontology) Ontology term synonyms |
| **Organism** | (InterMine) Organism information |
| **Orthologue Evidence** | (Comparative Genomics) Orthologue evidence |
| **Orthologue Evidence Code** | (Comparative Genomics) Orthologue evidence code |
| **Protocol** | (Omics) Protocol information |
| **Publication** | (Literature) Publication information |
| **Sequence** | (Genomics) Sequence information |
| **Sequence Variant** | (Genomics) Sequence Ontology term |
| **Submission Data** | (Omics) Container of submitted data per applied protocol |
| **Submission Property** | (Omics) Base class of all omics specific data types |
| **Antibody** | (Omics) Antibody information |
| **Data Analysis** | (Omics) Data analysis information |
| **Experimental Factor** | (Omics) Experimental feature information e.g. temperature |
| **Microarray** | (Omics) Microarray information |
| **Persistent Data File** | (Omics) Common data files e.g. POS for Nimblegen microarray |
| **Sequencing** | (Omics) Sequencing information |
| **Submission Data File** | (Omics) User submitted data file |
| **Toxoplasma Mutant** | (Omics) Toxoplasma mutant information |
| **Synonym** | (Ontology) synonym |
| **Uniprot Feature** | (Proteins) Uniprot features |
